# Prevalence and Determinants of Food Insecurity among United States Cancer Survivors Based on the Medical Expenditure Panel Survey 2021

**DOI:** 10.1158/2767-9764.CRC-25-0344

**Published:** 2026-04-10

**Authors:** Sisinyous Z. Mengesha, Anas K. Nabil, Mohammad A. Karim

**Affiliations:** 1 Department of Healthcare Administration and Policy, School of Public Health, University of Nevada, Las Vegas, Las Vegas, Nevada.; 2 Department of Health Behavior, School of Public Health, Texas A&M University, College Station, Texas.

## Abstract

Food insecurity is a significant public health issue that adversely affects the health outcomes of cancer survivors in the United States. This study estimated the prevalence of food insecurity among cancer survivors and identified its determinants in the year following the year of the COVID-19 outbreak. This cross-sectional study used data from the nationally representative Medical Expenditure Panel Survey (MEPS) 2021, which collected information on respondents’ health conditions (including history of cancer), healthcare utilization, sociodemographic characteristics, access to food, etc. This study included 2,500 adult respondents from MEPS 2021 who reported a history of cancer. The main outcome measure was food insecurity, which was defined based on responses to MEPS survey items regarding concerns about running out of food and the inability to afford food. Covariates included age, sex, race/ethnicity, marital status, educational attainment, poverty status, insurance status, and the number of comorbid conditions. The prevalence of food insecurity among cancer survivors was 13.47% overall in 2021. Younger cancer survivors (ages 18 to <50 years), those of non-Hispanic Black or non-Hispanic Asian/other race/ethnicity, those who were not married, had a high school diploma, had income <400% of federal poverty level, were <65 years with public only insurance or 65+ years with Medicare, and other public only insurance coverage demonstrated higher likelihood of experiencing food insecurity compared with their respective counterparts. Our findings can inform policy efforts to ensure access to nutritious food for the most vulnerable cancer survivors during times of economic volatility and recovery, as experienced in the year after the COVID outbreak.

**Significance:**

In this study using the nationally representative MEPS 2021 data, we found that more than 13% of cancer survivors experienced food insecurity in the year following the year of the COVID-19 outbreak, whereas certain subgroups, including younger and lower-income individuals, were especially vulnerable.

## Introduction

In the United States, cancer is the second most common cause of death overall, with more than 618,000 estimated deaths occurring in 2025 (1). The number of individuals living with a history of cancer is also substantial, with an estimated 18.6 million cancer survivors alive in the United States in 2025 (2). Cancer survivors frequently experience adverse physiologic impacts related to their condition and treatments, including diminished health-related quality of life and inability to perform daily tasks (3), which are compounded by the financial distress and material hardship experienced by them (4–7). Studies have consistently found higher financial distress among cancer survivors compared with individuals without a history of cancer (6, 7). This affects cancer survivors of all ages, as reported in a 2019 study in which 43.4%, 32.8%, and 17.3% survivors in the 18 to 49 years, 50 to 64 years, and ≥65 years age groups, respectively, reported having experienced material hardship (vs. 30.1%, 27.8% and 14.7% among the respective counterparts without a history of cancer; ref. 7). A major facet of material hardship experienced by the cancer survivors in the United States is food insecurity (8). Food insecurity, as defined by the US Department of Agriculture (USDA), is a “household-level economic and social condition of limited or uncertain access to adequate food” (9). Although food insecurity is not a problem unique to cancer (10–12), it is considered an important social needs parameter of health among cancer survivors (13, 14) because cancer causes substantial life-course disruptions making the survivors especially vulnerable. Due to its broad impact on health outcomes, food insecurity is an important aspect of cancer survivorship, and research shows that it is associated with higher incidence and mortality burden of cancer (15). Moreover, lower quality of food intake and reduced access to nutritious food increases the risk of cancer recurrence and overall mortality (16, 17).

Several previous studies reported food insecurity among cancer survivors; however, many of them used non–national-level data or pre–coronavirus disease (COVID)-19 national-level data (18). In the national-level studies published between 2015 and 2020, prevalence of food insecurity ranged between 8.4% to 26.2% among cancer survivors (18), whereas among the general US population, the prevalence was between 10.5% to 12.7% during the same time period according to USDA estimates (19). Worries about food running out was higher among cancer survivors compared with those without a history of cancer in the prepandemic period, as reported in a study using pooled 2013 to 2017 National Health Interview Survey (NHIS) data (20). The outbreak of COVID caused a sudden and drastic economic downturn in the United States in the beginning of the pandemic, causing substantial job and income losses in early 2020 (21). A few studies examined the extent of food insecurity among cancer survivors in the volatile economic environment at that time period. These studies examining the early months of COVID found that the pandemic was strongly linked to the exacerbation of health disparities in association with food insecurity and financial strain among many cancer survivors (22, 23). Camacho-Rivera and colleagues (23), examining data collected during the April 2020 to June 2020 period, found significantly higher prevalence of food insecurity among several subgroups of cancer survivors compared with those without a history of cancer. Certain subgroups were severely affected, with about 60% of the younger cancer survivors (ages 30–44 years) reporting food insecurity in that study (vs. 41.1% among those without a history of cancer in the same age group; ref. 23). The year that followed (2021) was characterized by early dissemination of COVID vaccines (24), financial assistance initiatives by the government (25), and early signs of economic recovery (26). The persistence and extent of the food insecurity problem among cancer survivors in that period of early economic recovery remains largely underexplored. To better address the challenges of food insecurity among cancer survivors in the post-COVID world, a deeper understanding of the changing dynamics of this problem during the gradually improving economic environment in the period following the early pandemic months is warranted. These insights may inform need-based policy interventions which may potentially help counter the issue of food insecurity among cancer survivors in times of economic recovery in the future. Government programs play a vital role in providing food assistance supports, and the Supplemental Nutrition Assistance Program (SNAP) has been reported to improve food quality and caloric intake (27) and reduce food insecurity among the recipients (28). Additionally, state public assistance spending has been associated with improved survival among cancer survivors (29). Future tailored approaches to adapt these programs to the food security needs of cancer survivors in economically volatile and recovery situations will warrant policy actions, which can be better informed with a deeper understanding of the changing landscape of food insecurity encountered in the year after the COVID outbreak. Our study addresses this critical issue through an investigation of a nationally representative data source in the United States, estimating the prevalence and identifying the determinants of food insecurity among cancer survivors in 2021.

## Materials and Methods

### Data source

In this cross-sectional study, we used data from the MEPS, 2021, which contains data collected in the calendar year 2021 (30). The MEPS, a survey sponsored by the Agency for Healthcare Research and Quality (AHRQ), is representative of the noninstitutionalized civilian population in the United States (30). In 2021, the overall response rate for the MEPS was 21.8% (30). The specific dataset used in this study, the Full Year Consolidated (FYC) file, is part of the Household Component of the MEPS, which is collected by a third party, Westat, Inc., under contract with the AHRQ. FYC data provides a broad range of information, including the history of cancer diagnosis, comorbid conditions, and sociodemographic characteristics of the respondents, including access to food (30).

### Study sample

For the purpose of this study, we identified cancer survivors in MEPS 2021 using the survey question that asked whether the survey respondent “had ever been diagnosed as having a cancer or malignancy of any kind” (30). The respondents answering “yes” to this question were ascertained to have had a history of cancer. Among them, those respondents with complete information for all the study variables were included in our study sample.

### Outcome variable: food insecurity

Following literature precedence, we identified the outcome variable, food insecurity, using the MEPS survey items “during last 12 months, worried about food running out before person got money to buy more” and “during last 12 months, food bought did not last and person did not have money to get more” (23, 30, 31). Respondents answering “often true” or “sometimes true” to either of these two items were considered to have experienced food insecurity in the 12-month period preceding the survey.

### Covariates

Pertinent variables selected *a priori* following literature precedence (23) were used as covariates in our adjusted statistical analyses (32). Covariates included age [18 to <50 years, 50 to <65 years (reference), 65 to <75 years, and ≥75 years], sex [male (ref.) and female], race/ethnicity [non-Hispanic White (ref.), non-Hispanic Black, Hispanic, and non-Hispanic Asian/other], marital status [married (ref.) and not married], educational attainment [<high school diploma, high school diploma, and college education or higher (ref.)], poverty status [<125% of the federal poverty level (FPL), 125% to <200% FPL, 200% to <400% FPL, and ≥400% FPL (ref.)], insurance status [<65 years with any private insurance (ref.), <65 years with public only insurance, 65+ years with Medicare only, 65+ years with Medicare and private insurance only, 65+ years with Medicare and other public insurance only, and 65+ years with no Medicare/any age uninsured], and number of MEPS priority conditions [none (ref.), one, two, and three or more]. We were interested in assessing how the experience of food insecurity among cancer survivors in the lower socioeconomic status compared with those in the higher socioeconomic status. As we did not construct a summary socioeconomic status variable, the levels representing the highest socioeconomic status in the poverty status (i.e., ≥400% FPL) and insurance status (i.e., <65 years with any private insurance) variables were set as the reference categories for each of these variables, respectively. In the marital status variable, all individuals who responded that they were divorced, separated, widowed, or were never married were included in the “not married” category. In the insurance status variable, respondents who were 65+ years with no Medicare coverage were combined with those without any insurance coverage (any age) to create the “65+ years with no Medicare/any age uninsured” category due to the small sample size in each constituent category. Comorbid conditions, presented in the MEPS with the term “priority conditions,” were categorized based on the actual number of conditions reported by the respondents. Included comorbid conditions were hypertension, heart disease, stroke, emphysema, chronic bronchitis, high cholesterol, diabetes, joint pain, arthritis, and asthma.

### Analysis

Descriptive statistics were used to characterize the study sample in terms of weighted number of observations, weighted percentage, and weighted prevalence of food insecurity, while accounting for the complex survey design of the MEPS (30). The MEPS uses oversampling of certain population subgroups to increase sample size and precision of estimates; hence, it is necessary to apply appropriate survey weights and design to obtain population-level estimates (30). We used multivariable logistic regression to identify the determinants of food insecurity, with appropriate survey weights and design adjustments applied. All statistical analyses were conducted in SAS 9.4 (RRID: SCR_008567) and Stata 18.5 (RRID: SCR_012763) at a two-sided 0.05 significance level (33, 34).

## Results

### Characteristics of the study sample

Of the 2,500 respondents with a history of cancer included in our study, 211 (11.83%) were ages 18 to <50 years, 879 were 75 years or older (32.59%), and 1,464 (57.39%) were female. In terms of race/ethnicity, 2005 (83.46%) were non-Hispanic White, 221 (6.45%) were non-Hispanic Black, 195 (6.76%) were Hispanic, and 79 (3.34%) were non-Hispanic Asian/other. The majority of the respondents had a college education or a higher degree (*n* = 1,249; 53.01%), and about half of the respondents (*n* = 1,025, 49.36%) had income ≥400% of the FPL. Respondents <65 years with any private insurance constituted 30.60% (*n* = 548), <65 years with public only insurance constituted 7.68% (*n* = 218), and 65+ years with Medicare and other public only insurance constituted 6.21% (*n* = 228) of the study sample. More than half of the respondents had a high comorbidity burden, with 1,435 (52.93%) reporting three or more MEPS priority conditions (Table 1; all reported percentages are weighted percentages).

**Table 1.** Sample characteristics: adult individuals with a history of cancer, MEPS, 2021^a^.

Variable	*n*	Weighted *n*	Weighted %	Prevalence of food insecurity, weighted %
Total	2,500	28,213,173	100	13.47
Age group	​	​	​	​
18 to <50 years	211	3,336,323	11.83	30.53
50 to <65 years	596	7,752,767	27.48	15.88
65 to <75 years	814	7,928,180	28.10	12.81
≥75 years	879	9,195,903	32.59	5.80
Sex	​	​	​	​
Male	1,036	12,021,115	42.61	10.79
Female	1,464	16,192,058	57.39	15.45
Race/ethnicity	​	​	​	​
Non-Hispanic White	2,005	23,546,969	83.46	10.32
Non-Hispanic Black	221	1,818,406	6.45	32.22
Hispanic	195	1,905,955	6.76	25.68
Non-Hispanic Asian/other	79	941,844	3.34	31.27
Marital status	​	​	​	​
Married	1,261	16,778,655	59.47	8.23
Not married^b^	1,239	11,434,518	40.53	21.15
Educational attainment	​	​	​	​
<High school diploma	204	1,753,650	6.22	31.35
High school diploma	1,047	11,503,202	40.77	17.52
College education or higher	1,249	14,956,322	53.01	8.25
Poverty status	​	​	​	​
<125% FPL	441	3,341,318	11.84	32.28
125% to <200% FPL	350	3,424,943	12.14	24.35
200% to <400% FPL	684	7,520,021	26.65	15.29
≥400% FPL	1,025	13,926,892	49.36	5.29
Insurance status	​	​	​	​
<65 any private	548	8,479,858	30.06	12.54
<65 public only	218	2,167,891	7.68	45.45
65+ Medicare only	659	6,612,579	23.44	8.20
65+ Medicare and private only	790	8,507,512	30.15	5.30
65+ Medicare and other public only	228	1,752,516	6.21	30.23
65+ with no Medicare/any age uninsured^c^	57	692,817	2.46	32.91
Number of MEPS priority conditions	​	​	​	​
None	166	2,547,036	9.03	13.98
One	390	4,966,722	17.60	10.74
Two	509	5,766,158	20.44	10.54
Three or more	1,435	14,933,257	52.93	15.41

a All weighted descriptive statistics are survey weight- and design-adjusted.

b All individuals responding that they were never married or were widowed, divorced, or separated were included in the “not married” category.

c Respondents who were 65+ years with no Medicare coverage were combined with those without any insurance coverage (any age) to create the “65+ years with no Medicare/any age uninsured” category due to the small sample size in each constituent category.

### Prevalence of food insecurity

The prevalence of food insecurity was 13.47% among all respondents with a history of cancer in our study. Food insecurity varied substantially among sociodemographic groups, with several groups of cancer survivors experiencing higher than 25% prevalence. Prevalence of food insecurity was 30.53% among cancer survivors ages 18 to <50 years, 31.35% among those with no high school diploma, and 32.28% among those with income <125% of the FPL. Among non-Hispanic Black, Hispanic, and non-Hispanic Asian/other cancer survivors, prevalence of food insecurity was 32.22%, 25.68%, and 31.27%, respectively. Across insurance status, 45.45% of the <65 years old cancer survivors with public only insurance and 30.23% of the 65+ years cancer survivors with Medicare and other public only insurance experienced food insecurity (Table 1; all reported percentages are weighted percentages).

### Determinants of food insecurity

In our adjusted logistic regression analysis, several factors were associated with food insecurity among cancer survivors. Compared with the respondents ages 50 to <65 years, those ages 18 to <50 years had higher odds [adjusted odds ratio (aOR), 2.66; *P* value < 0.001], and those ages ≥75 years had lower odds [aOR, 0.08; *P* value = 0.014] of having experienced food insecurity. Respondents who were of non-Hispanic Black (aOR, 2.63) or non-Hispanic Asian/other race/ethnicity (aOR, 3.38; both vs. non-Hispanic White; *P* value < 0.001 for both); were not married (vs. married; aOR, 1.86; *P* value < 0.001); had a high school diploma (vs. college education or higher; aOR, 1.60; *P* value = 0.009); had income <125% of the FPL (aOR, 3.15), 125% to <200% of the FPL (aOR, 3.84), or 200% to <400% of the FPL (aOR, 2.37; all vs. ≥400% of FPL; *P* value < 0.001 for all); were <65 years with public only insurance (vs. <65 years with any private insurance; aOR, 2.04; *P* value = 0.008) or were 65+ years with Medicare and other public only insurance (vs. <65 years with any private insurance; aOR, 8.37; *P* value = 0.038); or had three or more comorbid conditions (vs. none; aOR, 2.33; *P* value = 0.01) had higher odds of experiencing food insecurity (Table 2).

**Table 2.** Factors associated with food insecurity among US adults with a history of cancer^a^.

Variable	aOR (95% CI)	*P* value
Age group	​	​
**18 to <50 years**	**2.66 (1.55–4.55)**	**<0.001**
50 to <65 years	ref.	​
65 to <75 years	0.22 (0. 03–1.60)	0.133
**≥75 years**	**0.08 (0. 01–0.59)**	**0.014**
Sex	​	​
Male	ref.	​
Female	1.09 (0.79–1.51)	0.583
Race/ethnicity	​	​
Non-Hispanic White	ref.	​
**Non-Hispanic Black**	**2.63 (1.64–4.23)**	**<0.001**
Hispanic	1.66 (0.91–3.01)	0.096
**Non-Hispanic Asian/other**	**3.38 (1.76–6.48)**	**<0.001**
Marital status	​	​
**Married**	ref.	​
Not married^b^	**1.86 (1.32–2.61)**	**<0.001**
Educational attainment	​	​
<High school diploma	1.58 (0.82–3.07)	0.173
**High school diploma**	**1.60 (1.12–2.28)**	**0.009**
College education or higher	ref.	​
Poverty status	​	​
**<125% FPL**	**3.15 (1.95–5.08)**	**<0.001**
**125% to <200% FPL**	**3.84 (2.33–6.33)**	**<0.001**
**200% to <400% FPL**	**2.37 (1.51–3.70)**	**<0.001**
≥400% FPL	ref.	​
Insurance status	​	​
<65 any private	ref.	​
**<65 public only**	**2.04 (1.21–3.45)**	**0.008**
65+ Medicare only	3.35 (0.46–24.40)	0.232
65+ Medicare and private only	2.37 (0.32–17.55)	0.397
**65+ Medicare and other public only**	**8.37 (1.12–62.31)**	**0.038**
65+ with no Medicare/any age uninsured^c^	2.67 (0.96–7.43)	0.061
Number of MEPS priority conditions	​	​
None	ref.	​
One	1.28 (0.61–2.68)	0.513
Two	1.39 (0.70–2.74)	0.345
**Three or more**	**2.33 (1.23–4.40)**	**0.010**

a Results from the logistic regression model that takes account of the survey weight and design of the MEPS, 2021. Boldface indicates statistical significance (*P* < 0.05).

b All individuals responding that they were never married or were widowed, divorced, or separated were included in the “not married” category.

c Respondents who were 65+ years with no Medicare coverage were combined with those without any insurance coverage (any age) to create the “65+ years with no Medicare/any age uninsured” category due to the small sample size in each constituent category.

## Discussion

Food insecurity among cancer survivors is a concerning issue that adversely affects survivorship. In this study, we estimated the prevalence of food insecurity and identified its determinants among cancer survivors in the year after the COVID-19 outbreak year. We found that more than 13% of cancer survivors experienced food insecurity in 2021, whereas certain subgroups, including younger, minority, and lower-income individuals, were especially vulnerable.

Research suggests that food insecurity is integrally related to a higher risk of overall mortality among cancer survivors (35). Financial hardship and reallocating resources associated with persistent food insecurity are some of the factors that negatively affect cancer treatment outcomes among respective patients (8, 36, 37). Thus, food insecurity among cancer survivors demands close scrutiny, especially in the changing landscape following the COVID outbreak. Pre-COVID national estimates of food insecurity among cancer survivors varied substantially, ranging from 8.4% to 26.2% (18), likely due to variations in study samples, whereas it was reported to be around 20% within the first few months of COVID outbreak (April 2020–June 2020; ref. 23). Our 2021 overall prevalence estimate is within the pre-COVID range, indicating that food insecurity continued to be a pressing issue affecting cancer survivors in the year after the outbreak, following the surge in the early months of COVID.

Several previous studies reported association between food insecurity and demographic factors, including age and race/ethnicity, among cancer survivors. Individuals in the younger age groups are more likely to be food insecure and suffer from a lack of community health nutrition and food support when affected by cancer (32). According to a study by Zheng and colleagues (20) using NHIS data, younger cancer survivors ages 18 to 39 years demonstrated higher food insecurity during the pre-COVID period (2013–2017), with 7.9% frequently experiencing food running out and 7.6% often struggling to afford balanced meals, compared with 4.6% and 3.4%, respectively, among those without a cancer history. Our study found much higher prevalence of food insecurity among cancer survivors in a similar age range (18 to <50 years) in 2021, with 30.5% reporting food insecurity. We also found that survivors in this age group (18 to <50 years) had more than 2.6 times higher odds of experiencing food insecurity compared with those in the 50 to <65 years age group. Younger cancer survivors are more financially vulnerable because of their dependence on employment-related health insurance coverage and income, both of which can be disrupted with a cancer diagnosis (7). The sudden rise in unemployment in the early months of COVID, which was yet to fully recover in 2021, exacerbated this situation, which is a potential reason behind the high food insecurity prevalence observed among the younger survivors in our study.

Similarly, we found higher likelihood of food insecurity among minority groups in 2021, with non-Hispanic Black and non-Hispanic Asian/other cancer survivors demonstrating statistically significant higher odds of experiencing food insecurity compared with non-Hispanic White survivors. In our findings, prevalence of food insecurity was higher than 25% among non-Hispanic Black, Hispanic, and non-Hispanic Asian/other cancer survivors (vs. 10.32% among non-Hispanic White survivors), which is in line with the prepandemic estimates for the minority survivor groups (32). In a study, analyzing 2011 to 2014 National Health and Nutrition Examination Survey data, Trego and colleagues (32) found prevalence of food insecurity in the similar range among non-Hispanic Black (20.60%), Mexican American (30.46%), and other Hispanic (25.49%) cancer survivors (vs. 6.25% among non-Hispanic White survivors). In contrast to the prepandemic estimates, early pandemic estimates of prevalence of food insecurity among minority cancer survivors are much higher than our estimates across similar groups (23). Analyzing COVID-19 impact survey data, Camacho-Rivera and colleagues (23) found that about half of the non-Hispanic Black (50.9%) and Hispanic (49.6%) cancer survivors experienced food insecurity during the April 2020 to June 2020 period (vs. 25.6% among non-Hispanic White survivors). Higher prevalence of food insecurity compared with the pre-COVID period and lower prevalence compared with the early-COVID period across these groups in our study is likely a reflection of the exacerbated financial conditions faced by minorities during the onset of the COVID-19 pandemic (22), which gradually improved over time. The second and third rounds of the COVID-related economic impact payments (stimulus checks) were paid in 2021 (25), and the job market started to gradually recover in 2021, after the unprecedented level of job cuts in 2020 (21). These positive developments may have contributed in improved financial conditions in 2021, which is potentially a reason behind the reduced prevalence of food insecurity among cancer survivors in 2021 compared with early 2020. Job loss is a persistent issue affecting cancer survivors’ financial conditions (38), and several studies indicate that COVID exacerbated the problem (39, 40). However, our study was not designed to detect the direct impact of COVID related job and income loss on food insecurity. Future research should focus on this to understand how a public health emergency may affect these relationships among cancer survivors and how the conditions might change—and potentially improve—during the period of recovery that follows.

Marital status affects financial hardship and care seeking among cancer survivors (41). Previous studies reported that cancer survivors experiencing financial hardship are more likely to be unmarried (42). Our study found that access to adequate food among this subgroup of survivors is also affected, with unmarried cancer survivors reporting nearly 1.8 times higher odds of experiencing food insecurity compared with their married counterparts in 2021. This indicates a broader challenge experienced by unmarried cancer survivors who may have faced accessibility issues in terms of both medical care and healthy food during 2021.

Family income is another important factor that directly affects food insecurity. As Zheng and colleagues (20) found in an analysis of pre-COVID data (2013–2017 NHIS), lower income was associated with higher intensities of food insecurity among cancer survivors. Consistent with that pre-COVID report, we found that cancer survivors in families with income <400% of the FPL were more likely to experience food insecurity in the year after the COVID outbreak year.

Moreover, the relationship between poverty conditions and food insecurity is closely linked to health insurance coverage (43, 44), with previous works proposing a cyclical relationship among these factors (32, 45, 46). When individuals have limited spending abilities, it may cause reduced spending on healthy foods, which may result in their worse health status. This, consequently, may fuel higher healthcare spending, reducing spending abilities on other necessities, thus propagating the cyclic relationship (46). Lack of adequate health insurance may further strain an individual’s spending abilities, often resulting in delayed or forgone health service utilization (47), which may worsen this detrimental cycle. Additionally, individuals with public insurance—such as Medicaid—often experience higher financial distress compared with those with private insurance coverage (48). Cancer survivors are not immune to this cycle, and those who do not have adequate insurance coverage or rely on public insurance programs could be disproportionately affected by financial distress and food insecurity. This compounded impact is evident in our study, in which individuals younger than 65 years with public only insurance and 65+ years with Medicare and other public only insurance demonstrated significantly higher likelihood of food insecurity. This finding highlights the need for targeted support for these groups of cancer survivors during economic recovery periods. Established food aid programs, such as the SNAP, can potentially play a crucial role in addressing food insecurity among cancer survivors (28, 49). Access to the SNAP can provide adequate caloric intake and improve the quality of food (27), reduce food insecurity (28), and reduce health-related spending (50). Furthermore, state public assistance spending has been reported to improve overall survival among cancer survivors (29). A combination of SNAP and Medicaid coverage could provide a sustainable safety net by meeting nutritional food demands for cancer recovery, improving access to food products, and enhancing dietary quality. Therefore, future interventions studies should be designed to assess the combined impact of SNAP and Medicaid among cancer survivors, especially among those who have historically been considered medically underserved and likely experience a high risk of food insecurity (18).

Food insecurity is not a unique problem among cancer survivors, as reported in previous studies and also observed in our analysis (Supplementary Table S1). Individuals with various health conditions, including cardiovascular disease (51), diabetes (52), chronic kidney disease (10), chronic liver disease (11), and asthma (12), are affected by food insecurity. However, in comparison with other major chronic health conditions, cancer survivorship trajectories often show profound life-course disruptions. On one hand, there are chronic disease-specific stressors such as treatment-related physiologic and psychologic complications (53); on the other hand, there are financial uncertainty and anxiety of disease recurrence that affect cancer survivors (54). Our analysis provides insights into the interplay between cancer and other comorbidities in the year following the COVID outbreak year, which found that increased number of comorbidities substantially increased food insecurity among cancer survivors. The odds of experiencing food insecurity more than doubled when there were three or more comorbid conditions present besides cancer. Thus, our findings suggest that the inherent challenges of food insecurity associated with cancer were compounded by the presence of comorbidities.

### Limitations

There were several limitations in our study. First, the study may not fully represent all cancer survivors because institutionalized and noncivilian populations were beyond the scope of this study. However, these missed population segments are fairly small in size to substantially affect the study outcomes. Second, survey participants tend to have a self-selection bias and are generally healthier than nonparticipants (55), which may introduce bias in the study estimates. Third, due to sample size constraints, it was not possible to conduct subgroup analyses by cancer type in this study. Fourth, food insecurity was identified using two self-reported survey questions, not with a standardized measurement instrument, which may result in improper identification of food insecurity and may be associated with recall bias (56). Fifth, this was a cross-sectional study, which was not designed to capture the temporal trend in food insecurity. Moreover, response rate for the MEPS dropped substantially due to COVID, dropping from 39.5% in 2019 to 27.6% in 2020 and 21.8% in 2021, respectively, which may skew the data. The racial/ethnic composition of the cancer survivors in our study with respect to the survivors at the population level—although very close—was not exactly the same. Despite these limitations, the broad nationally representative nature of the MEPS provided us with a valuable tool to investigate food insecurity affecting cancer survivors during the initial economic recovery period in 2021.

### Conclusion

Food insecurity is a key social needs parameter that adversely affects the life and wellbeing of cancer survivors. In this study, we found that more than 13% of the cancer survivors in the United States experienced food insecurity in the year following the year of the COVID outbreak. The prevalence was much higher among several sociodemographic groups, with younger, minority, and poorer cancer survivors demonstrating higher vulnerability. Our findings show that the prevalence of food insecurity among several sociodemographic groups of cancer survivors were lower than the respective levels previously reported for the early pandemic months (early 2020) and approached the prepandemic level in 2021. This indicates that the government interventions and job recovery during the early economic recovery period of 2021 may have positively affected the extent of food insecurity among cancer survivors. However, further research is warranted to gain a deeper understanding of this impact. To ensure adequate availability of nutritious food among the most vulnerable cancer survivors in a post-COVID world, and to better prepare for future periods of economic volatility and recovery, appropriately designed policy initiatives are warranted. Our findings can provide valuable insights to inform such policy initiatives.

## Supplementary Material

Supplementary Table S1 Table S1. Prevalence of food insecurity across comorbid conditions among adult individuals, Medical Expenditure Panel Survey, 2021 (N = 17914)

## Data Availability

Data used in this study are publicly available and can be obtained from the MEPS website maintained by the AHRQ (30).
