# Supplementary material for: Prevalence and Determinants of Food Insecurity among United States Cancer Survivors Based on the Medical Expenditure Panel Survey 2021
Source: Cancer Res Commun. 2026 Apr 10;6(4):803–10. doi: 10.1158/2767-9764.CRC-25-0344 (PMC13067244; doi:10.1158/2767-9764.CRC-25-0344)
Supplement: Supplementary Table S1 — Table S1. Prevalence of food insecurity across comorbid conditions among adult individuals, Medical Expenditure Panel Survey, 2021 (N = 17914) [file crc-25-0344_supplementary_table_s1_suppst1.docx]

| **Supplemental Table 1. Prevalence of food insecurity across comorbid conditions among adult individuals, Medical Expenditure Panel Survey, 2021 (N = 17914)** | | | | |
| --- | --- | --- | --- | --- |
| **Comorbid condition** | **n** | **Weighted n** | **Weighted %** | **Prevalence of food insecurity, weighted %** |
| Hypertension | 7136 | 81737323 | 33.21 | 19.84 |
| Heart disease | 3122 | 35516308 | 14.43 | 18.57 |
| Stroke | 927 | 9398654 | 3.82 | 27.49 |
| Emphysema | 398 | 3696987 | 1.50 | 31.37 |
| Chronic bronchitis | 377 | 3848614 | 1.56 | 40.9 |
| High cholesterol | 6711 | 77932246 | 31.66 | 17.82 |
| Diabetes | 2639 | 27713060 | 11.26 | 24.2 |
| Joint pain | 3811 | 54030392 | 21.95 | 19.61 |
| Arthritis | 5729 | 62683741 | 25.47 | 20.94 |
| Asthma | 2723 | 35403642 | 14.38 | 26.78 |

Note: Comorbid conditions are not exclusive and each may overlap with other comorbidities and cancer. All weighted descriptive statistics are survey weight and design adjusted.
